# Chromatin structure characteristics of pre-miRNA genomic sequences

**DOI:** 10.1186/1471-2164-12-329

**Published:** 2011-06-25

**Authors:** Shijia Zhu, Qinghua Jiang, Guohua Wang, Bo Liu, Mingxiang Teng, Yadong Wang

**Affiliations:** 1 Center for Biomedical Informatics, School of Computer Science and Technology, Harbin Institute of Technology, Harbin, Heilongjiang, 150001, China

## Abstract

**Background:**

MicroRNAs (miRNAs) are non-coding RNAs with important roles in regulating gene expression. Recent studies indicate that transcription and cleavage of miRNA are coupled, and that chromatin structure may influence miRNA transcription. However, little is known about the relationship between the chromatin structure and cleavage of pre-miRNA from pri-miRNA.

**Results:**

By analysis of genome-wide nucleosome positioning data sets from human and *Caenorhabditis elegans *(*C. elegans*), we found an enrichment of positioned nucleosome on pre-miRNA genomic sequences, which is highly correlated with GC content within pre-miRNA. In addition, obvious enrichments of three histone modifications (H2BK5me1, H3K36me3 and H4K20me1) as well as RNA Polymerase II (RNAPII) were observed on pre-miRNA genomic sequences corresponding to the active-promoter miRNAs and expressed miRNAs.

**Conclusion:**

Our results revealed the chromatin structure characteristics of pre-miRNA genomic sequences, and implied potential mechanisms that can recognize these characteristics, thus improving pre-miRNA cleavage.

## Background

MicroRNAs (miRNAs) are a class of approximate 22-nt non-coding RNAs capable of binding to the 3' UTR of their target genes, resulting in transcriptional destabilization and/or post-transcriptional inhibition [1,2]. Typically, miRNA biogenesis initiates at transcription by RNA Polymerase II (RNAPII) [3], and produces a primary transcript (pri-miRNA). A canonical pri-miRNA is composed of a stem, a terminal loop, and long flanking sequences. Next, pri-miRNA is cleaved by a class 2 RNase III named Drosha (and its cofactor DGCR8) to yield a precursor miRNA (pre-miRNA) with a characteristic hairpin structure. The Drosha-DGCR8 complex is termed as Microprocessor [4].

Eukaryotic genomes are packaged together with nucleosomes making up chromatin. This organization embodies two distinct structures: sequence and chromatin structure. At the aspect of sequence, the pri-miRNAs are quite diverse. For example, different sites of stem in pri-miRNA may have different numbers of unpaired nucleotides, and the length of terminal loop by bioinformatic prediction could range from 3-nt to more than 10-nt [5-7]. It is also worth noting that no common sequence motif associated with human pri-miRNAs has been discovered [8]. Thus, a confusing issue is introduced: how can these diverse RNA structures be identified by the same complex and precisely cleaved to derive pre-miRNA. Recent studies indicate that the double-stranded stem and the unpaired flanking regions are crucial for the recognition and cleavage by Microprocessor [8-10]. However, the factors which contribute to the recognition of pre-miRNA genomic sequences are still far from being well-understood. Duan et al. indicated that the Drosha processing can be hindered by a single nucleotide polymorphism emerging in the stem of pri-miRNAs [11], whereas by studying the tumor-associated mutation, Diederichs et al. found that the Drosha processing remains intact even if the secondary structure of pri-miRNA is altered by many sequence variations found in human cancers [12]. These facts highlight the flexibility of the pre-miRNA recognition.

Chromatin structure, mainly concerned with how and where the DNA is packaged around nucleosomes, is affected by chemical modifications of histone proteins, including acetylation, methylation, phosphorylation and ubiquitination [13]. Saito et al. showed that some of these histone modifications can alter the miRNA expression level [14]. Also, Scott et al. found that treatment with HDAC inhibitor (stabilizing histone acetylation) can result in a rapid change in miRNA expression [15]. Recently, researchers employed transcriptionally active features to systematically identify promoters for known miRNA genes [16-18]. Exploiting these predicted miRNA promoters, Barski et al. found that histone modifications create a chromatin environment that can modulate transcriptional activation of miRNAs [18].

Traditionally, the sequence and chromatin structure are thought to function separately, sequence (primary transcript) at the RNA level, and chromatin structure at the DNA level. However, accumulating evidence suggests that processes occurring at these two levels are coupled. Recent research suggests that Drosha cleavage of pre-miRNAs from pri-miRNAs occurs co-transcriptionally, for both independently transcribed and intron-located miRNAs [19]. Also, Jan et al. observed that the pri-miRNAs retained at transcription sites are processed more efficiently into pre-miRNAs than the released pri-miRNAs, and the efficiently processed pri-miRNAs are enriched in chromatin-associated regions [20]. The results suggest that pri-miRNA processing is influenced by the chromatin structure when coupled with transcription. Recently, genome-wide data for nucleosome positioning and several epigenetic histone modifications have been released [21-23]. These data enable us to assess the distribution of nucleosome and histone modifications along pre-miRNA genomic sequences as well as their relationships to other genomic features. A pioneering study by Ozsolak et al. discovered an enrichment of nucleosome on pre-miRNA genomic sequence [16], suggesting that the pre-miRNA has specific chromatin characteristics. In this study, a series of statistical analyses about chromatin structure and related genomic features were performed, including nucleosome occupancy, specific epigenetic histone modifications, GC content, and RNAPII occupancy along pre-miRNA genomic sequences. Through these analyses, we found that the nucleosomes are enriched on all pre-miRNA genomic sequences corresponding to miRNAs experiencing different phrases within biogenesis (potentially transcribed, untranscribed, expressed and unexpressed miRNAs), and miRNAs located in different positions relative to protein-coding genes (intronic, intergenic, and exonic miRNAs). In addition, three histone modifications (H2BK5me1, H3K36me3 and H4K20me1) as well as RNAPII are obviously enriched along the pre-miRNA genomic sequences corresponding to potentially transcribed miRNAs and expressed miRNAs. The factors, which contribute to pre-miRNA recognition, are previously understood to only lie in the primary transcripts (RNA level), but our results suggest that they may also reside in the chromatin structure (DNA level), thus providing a new way to better appreciate the connection between chromatin structure, sequence and pre-miRNA cleavage.

## Results

### Nucleosome enrichment on pre-miRNA genomic sequence

**Figure 1:**
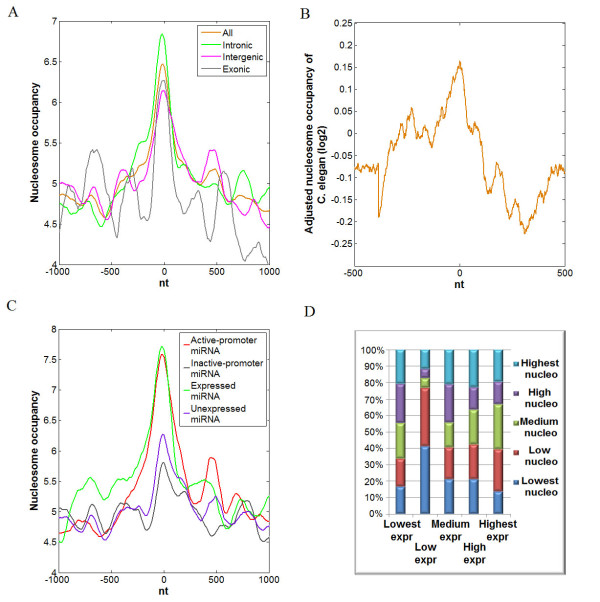
**Nucleosome is enriched within pre-miRNA genomic sequence**. The y axis represents the value of nucleosome occupancy. Position zero on X axis corresponds to the center of pre-miRNA genomic sequence. (A) Nucleosome occupancy profiles within 2,000-nt windows surrounding the centers of intronic, intergenic and exonic pre-miRNAs in resting CD4+ T cells. (B) The nucleosome occupancy profiles along the pre-miRNA genomic sequences in *C. elegans*. (C) Nucleosome occupancy profiles within 2,000-nt windows surrounding the centers of pre-miRNA genomic sequences corresponding to active-promoter miRNA, inactive-promoter miRNA, expressed miRNA and unexpressed miRNA. (D) Distribution of nucleosome occupancy in five groups of miRNAs with different expression levels. Five groups are defined according to the corresponding mature miRNA expression levels. Five different colors indicate the different levels of nucleosome occupancy in a 400-nt window surrounding the center of pre-miRNA.

To investigate the distribution of nucleosomes across pre-miRNA genomic sequence, we downloaded the annotation of 715 human pre-miRNAs from miRBase [24], and obtained a data set of genome-wide nucleosome positioning within the human resting CD4+ T cells. To assess whether nucleosomes are highly distributed within pre-miRNA genomic sequences, we examined the profiles of nucleosome occupancy across a 2,000-nt window surrounding the center of pre-miRNA genomic sequence. We found an obvious peak of nucleosome occupancy on pre-miRNA genomic sequence (Figure 1A, orange curve). This peak in pre-miRNA genomic sequence is significantly higher than the nucleosome occupancy of flanking regions (Wilcoxon signed-rank test p-value < 2.2e-12). The percentage of miRNAs with different levels of nucleosome occupancy is demonstrated in Additional file 1. Moreover, according to the location relative to protein-coding gene, miRNAs are categorized into intronic, intergenic and exonic miRNAs. The group annotation for each miRNA is presented in Additional file 2. To assess the influence of the relative positions to protein-coding gene, we examined the nucleosome occupancy profile along intronic, intergenic and exonic pre-miRNA genomic sequences, respectively (Figure 1A, green, pink and grey curves). We found that nucleosome occupancies in three kinds of pre-miRNA genomic sequences are significantly higher than those within flanking regions (Wilcoxon signed-rank test p-value < 2.2e-12 for intronic and intergenic, and = 3.616e-09 for exonic), however, as for exonic pre-miRNA, we also found high nucleosome occupancy in its flanking regions. This is probably because the exon is also occupied by nucleosome as indicated by recent studies [25,26]. In addition, we made comparison between the nucleosome occupancies on the genomic sequences of intronic, intergenic and exonic pre-miRNAs (details are seen in Methods), and found that none is statistically significantly higher than others (p-values = 0.06271 for comparison between intronic and intergenic pre-miRNAs, 0.134 for intronic and exonic, and 0.2976 for intergenic and exonic). This showed that nucleosome occupancy has no distinct association with the relative positions of miRNAs to protein-coding genes. Furthermore, to investigate whether the nucleosome enrichment on pre-miRNA is evolutionarily conserved, we assessed the high-throughput sequence data on nucleosome positioning in *Caenorhabditis elegans *(*C. elegans*) [27]. A peak of nucleosome occupancy was clearly seen on pre-miRNA genomic sequence (Figure 1B) (Wilcoxon signed-rank test p-value < 2.2e-12).

We derived the mature miRNA expression profile and putative miRNA promoters of CD4+ T cells from Ref. [18]. These promoters are predicted employing characteristic transcriptionally active features for genes (RNAPII, H3K4me3 and H2A.Z), which are indirect reflection of transcription. Based on predicted miRNA promoters and mature miRNA expression profiles, we derived four groups of miRNAs, including 230 active-promoter miRNAs, 243 inactive-promoter miRNAs, 206 expressed miRNAs and 329 unexpressed miRNAs (group definition details are seen in Methods). It is noted that overlaps were allowed between the categories to address the different steps during the miRNA biogenesis. The group annotation for each miRNA is presented in Additional file 2. The nucleosome occupancy is assessed in these four groups, respectively. As shown in Figure 1C, we discovered higher nucleosome occupancy on pre-miRNA genomic sequences corresponding to miRNAs of all three groups than their flanking regions (Wilcoxon signed-rank test p-value < 2.2e-12 for active-promoter, expressed and unexpressed miRNAs, and p-value = 3.761e-12 for inactive-promoter miRNAs), and found that the enrichments of nucleosome occupancy in active-promoter miRNAs and expressed miRNAs are significantly higher than the inactive-promoter miRNAs and unexpressed miRNAs (Wilcoxon signed-rank test p-value = 1.676e-06 for comparison between active-promoter and inactive-promoter, and p-value = 0.0001272 for expressed and unexpressed). In addition, we divided pre-miRNAs into five groups according to their corresponding mature miRNA expression levels: the pre-miRNAs are treated as being with "Lowest expression", whose expression levels are zero (no mature miRNAs found), and the remaining pre-miRNAs are divided into four groups with equal size. As shown in Figure 1D, nucleosome occupancies are almost uniformly distributed in each of the five groups. Furthermore, we evaluated the correlation between mature miRNA expression levels and corresponding nucleosome occupancies using Kendall's rank correlation test (tau = 0.1134140, p-value = 0.0004294). These facts suggest that there is no significant dependency between nucleosome occupancy and mature miRNA expression levels, consistently with the previous study in three human cancer cell lines [16]. A detailed list of miRNAs with regards to their expression profiles and nucleosome occupancies was presented in Additional file 2.

### Histone modification enrichment on human pre-miRNA genomic sequence

**Figure 2:**
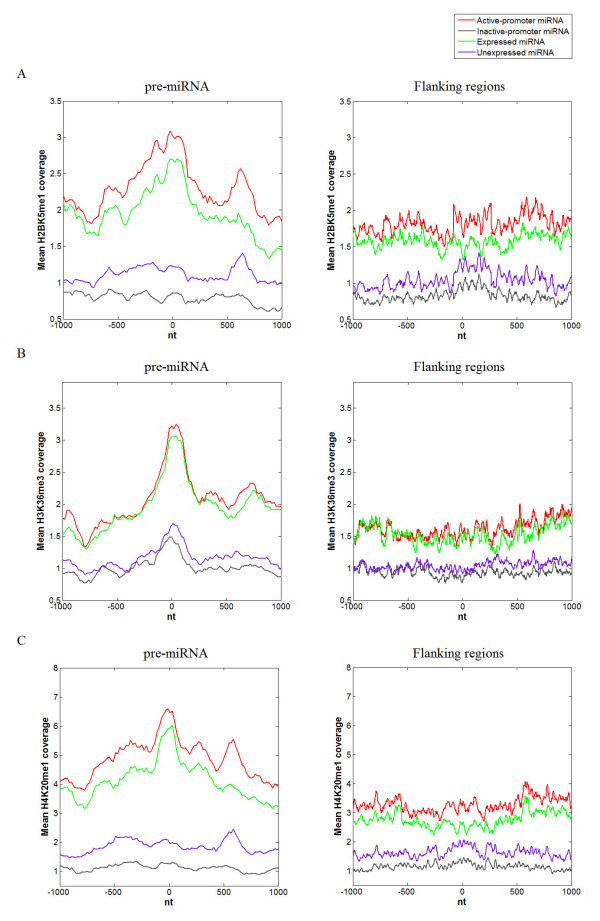
**Profiles of histone modification H2BK5me1, H3K36me3 and H4K20me1 along pre-miRNA genomic sequence in CD4+ T cells**. The y axis represents the mean value of histone modification coverage. Position zero on x axis corresponds to the center of pre-miRNA. (A) The occupancy profiles of H2K5me1 across a 2,000-nt window surrounding the centers of pre-miRNA genomic sequences corresponding to active-promoter miRNA, inactive-promoter miRNA, expressed miRNA and unexpressed miRNA (left) and those from their flanking regions (right). (B) The occupancy profiles of H3K36me3 across a 2,000-nt window surrounding the centers of pre-miRNA genomic sequences corresponding to active-promoter miRNA, inactive-promoter miRNA, expressed miRNA and unexpressed miRNA (left) and those from their flanking regions (right). (C) The occupancy profiles of H4K20me1 across a 2,000-nt window surrounding the centers of pre-miRNA genomic sequences corresponding to active-promoter miRNA, inactive-promoter miRNA, expressed miRNA and unexpressed miRNA (left) and those from their flanking regions (right).

Nucleosome is the region where histone modifications occur. When pre-miRNA genomic sequences are occupied by nucleosomes, specific histone modifications may also take place. A genome-wide ChIP-seq data set of 38 histone modifications of the human CD4+ T cells was downloaded from [22,23]. The average level of each histone modification was investigated in the pre-miRNA genomic sequences corresponding to active-promoter miRNAs, inactive-promoter miRNAs, expressed miRNAs and unexpressed miRNAs, respectively. Obvious enrichments of several histone modifications were observed within pre-miRNA genomic sequence, including H2BK5me1, H3K36me3 and H4K20me1 (Figure 2). These histone modifications have been previously reported as robust features of active genes [22,28]. Consistently, we also found that they have significantly higher distributions along pre-miRNA genomic sequences corresponding to active-promoter miRNAs and expressed miRNAs than inactive-promoter miRNAs and unexpressed miRNAs. Also, to assess whether the histone modifications are significantly different between pre-miRNA and its flanking regions, we calculated the nucleosome occupancy across the 2,000-nt window surrounding the center of each pre-miRNA genomic sequence, and the 2,000-nt windows selected from its flanking regions (details are seen in Methods). We found that they are all significantly higher within pre-miRNA genomic sequence than within their flanking regions (Wilcoxon signed-rank test p-value = 1.241e-13 for H2K5me1, p-value = 3.109e-15 for H3K36me3 and p-value = 3.553e-15 for H4K20me1).

### Effect of GC content on nucleosome occupancy

**Figure 3:**
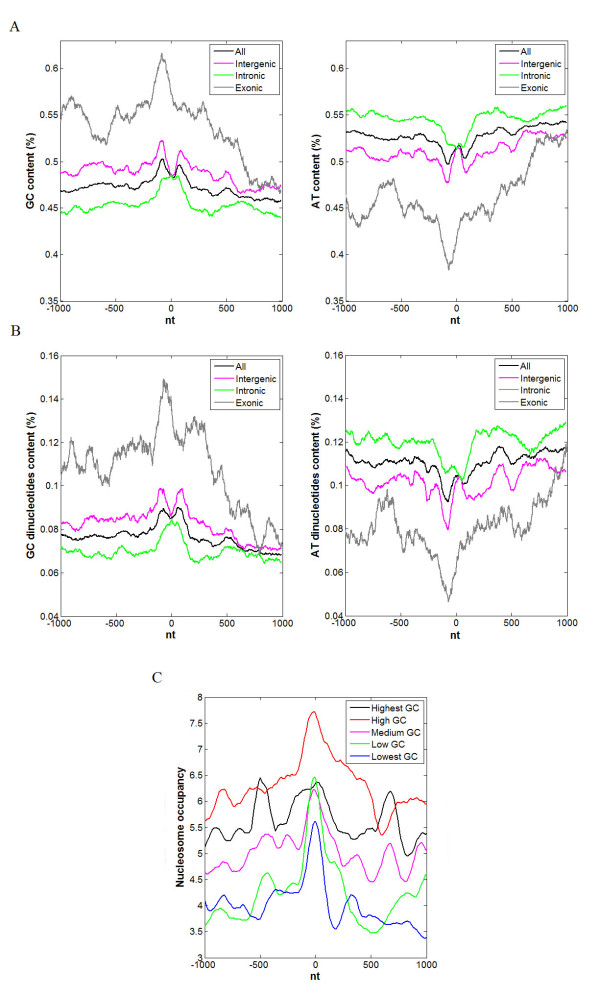
**Contents of GC, GC dinucleotides, AT, AT dinucleotides along pre-miRNA genomic sequence**. Position zero on X axis corresponds to the center of pre-miRNA. (A) The GC (left) and AT (right) contents across a 2,000-nt window surrounding the center of pre-miRNA genomic sequence. (B) The GC dinucleotides (left) and AT dinucleotides (right) contents across a 2,000-nt window surrounding the center of pre-miRNA genomic sequence. (C) Nucleosome occupancy in a 2,000-nt window surrounding the center of pre-miRNA genomic sequence. The pre-miRNAs are divided into five equal bins (lowest, low, medium, high and highest) according to the GC content in a 400-nt window surrounding the center of pre-miRNA genomic sequence.

Nucleosome occupancy is influenced by DNA sequence to a great extent [29-31]. Peckham et al. indicated that GC and AT are respectively the strongest factors to facilitate and inhibit the nucleosome formation, among all of the single k-mers [31]. Also, Johnson et al. found an enrichment of AT dinucleotides in linker DNA and an enrichment of GC dinucleotides in nucleosome core DNA [32]. In the section above, we have observed an enrichment of nucleosome occupancy within pre-miRNA genomic sequences, and therefore, we analyzed the contents of GC, GC dinucleotides, AT and AT dinucleotides to assess whether specific sequence characteristics exist within pre-miRNA genomic sequences. As shown in Figure 3A-B, we found that contents of GC, and GC dinucleotides within pre-miRNA genomic sequence are significantly higher than those in its surrounding sites (Wilcoxon signed-rank test p-value < 2.2e-12 for both two), and the contents of AT, and AT dinucleotides within pre-miRNA genomic sequence are significantly lower than in its surrounding sites (Wilcoxon signed-rank test p-value < 2.2e-12 for both two) (the black lines). Furthermore, to assess the dependence of relative positions of miRNAs to protein-coding genes, we also analyzed the GC/AT content across intronic, intergenic and exonic pre-miRNAs, respectively. Previous studies indicated that GC content in exons is significantly higher than that in introns [25,26]. Consistently, as shown in Figure 3A-B, both of GC and GC dinucleotides exert the highest levels along exonic pre-miRNA genomic sequence, and the lowest levels along intronic one; it is reversed for AT and AT dinucleotides. However, even if the distinct levels are observed, the significantly higher GC (lower AT) contents are shown in these pre-miRNA sequences with respects to their flanking regions (Wilcoxon signed-rank test p-value < 2.2e-12 for all). In addition, we found two peaks of GC content along pre-miRNA (Figure 3A-B (left)). By aligning the peaks to genome, we found that the two peaks place at the stem of miRNA hairpin, and the concaved part between the two peaks is at the terminal loop. However, we only found one peak for nucleosome positioning within pre-miRNA as shown in Figure 1. This is possibly because an average pre-miRNA is only approximately 70-nt long [33], whereas an average nucleosome cover approximately 147-nt [34], thus, these two peaks with short length may not tend to cover two nucleosomes.

To further investigate the correlation between DNA sequence and nucleosome occupancy, we divided pre-miRNAs into five equal bins according to the GC content in a 400-nt window surrounding the center of pre-miRNA genomic sequence, and examined the correlation between GC content and nucleosome occupancy (the details are in the method section). As shown in Figure 3C, nucleosome occupancy was increased with the elevated GC content on pre-miRNA genomic sequence, except the group with the highest GC content (60%~82%). This is similar to the observation on exons in human CD4+ T cells [25]. The facts above suggest that GC composition could be one of the factors that influence nucleosome occupancy on pre-miRNA genomic sequence.

### RNA Polymerase II enrichment on human pre-miRNA genomic sequence

**Figure 4:**
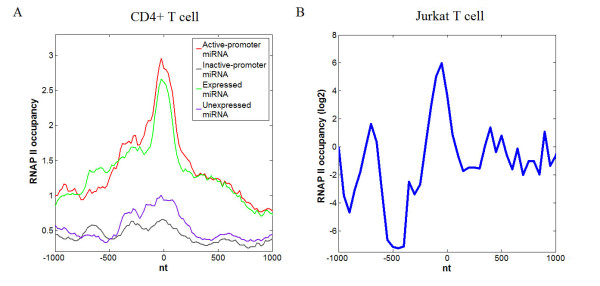
**RNAPII is preferentially positioned on pre-miRNA genomic sequence**. Position zero on X axis corresponds to the center of pre-miRNA genomic sequence. (A) The occupancy profiles of RNAPII across a 2,000-nt window surrounding the centers of pre-miRNA genomic sequences corresponding to active-promoter miRNA, inactive-promoter miRNA, expressed miRNA and unexpressed miRNA in CD4+ T cell. (B) The occupancy profiles of RNAPII across a 2,000-nt window surrounding the center of pre-miRNA genomic sequence in Jurkat T cell.

Several studies suggest that nucleosomes on exons could act as 'speed bumps' to facilitate exon splicing by reducing the rate of RNAPII [35-37]. Moreover, based on analysis of genome-wide data sets, increased binding levels of RNAPII are discovered within exons, compared to introns [25,26]. These increased levels are regarded as the consequence of reduction of RNAPII rate. Motivated by these results, we analyzed the genome-wide ChIP-seq data of RNAPII [22] within pre-miRNA genomic sequence. Significantly higher binding levels of RNAPII (Figure 4A) are obtained within pre-miRNA genomic sequences corresponding to active-promoter and expressed miRNAs, compared with flanking regions (Wilcoxon signed-rank test p-value < 2.2e-12). This is consistent with the observation on several specific miRNAs by Morlando et al. [19]. To further validate this enrichment, we also downloaded the datasets of RNAPII in Jurkat T cells (GSM479904) from Gene Expression Omnibus [38], analyzed the RNAPII occupancy along pre-miRNA genomic sequence, and discovered a similar increased RNAPII occupancy on pre-miRNA (Figure 4B). The results above suggest that the enrichment of RNAPII could result from reduction of its elongation rate caused by nucleosome bound on pre-miRNA genomic sequences. The fact that miRNA-encoding introns were spliced more slowly than the adjacent introns [39] partially validates the rate reduction of RNAPII passing by pre-miRNA.

**Figure 5:**
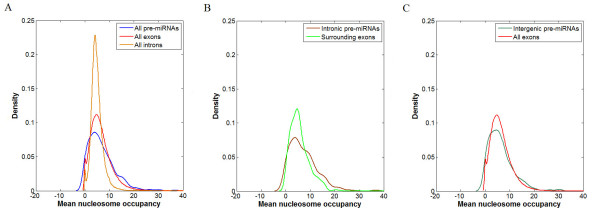
**Distribution of nucleosome occupancy in pre-miRNA, exon and intron**. (A) Density plots for all pre-miRNAs, exons and introns. These plots illustrate the frequencies of nucleosome positioning among pre-miRNAs and exons. (B) Density plots for all intronic pre-miRNAs and their two neighboring exons. (C) Density plots for all intergenic pre-miRNAs and exons.

Motivated by RNAPII elongation rate reduction caused by enrichment of nucleosome on exons, we made a comparison of the occupancies within all pre-miRNAs, exons and introns, and found that nucleosome occupancy within pre-miRNA genomic sequence is similar to that within exon and significantly higher than that within intron (Wilcoxon signed-rank test p-value < 2.2e-12) (Figure 5A). Also, we made a comparison between the nucleosome occupancy distribution in intronic pre-miRNA and its two neighboring exons, and did not find significant differentiation (Figure 5B). Furthermore, we looked at the nucleosome occupancy pattern within all exons and that within intergenic pre-miRNAs (Figure 5C), and still obtained the same results. Therefore, nucleosome within pre-miRNA could also function as a similar role to that within exons, i.e., serve as 'speed bumps' that slow the elongation rate of RNAPII.

## Discussion

Our analyses revealed that nucleosome occupancy is significantly higher in regions of the genome encoding pre-miRNAs of human CD4+ T cells with regards to their surrounding regions. The nucleosome enrichments are observed on all pre-miRNA genomic sequences corresponding to miRNAs experiencing different phrases within biogenesis (potentially transcribed, untranscribed, expressed and unexpressed miRNAs), and miRNAs located in different positions relative to protein-coding genes (intronic, intergenic, and exonic miRNAs). The similar enrichment of nucleosome is discovered in *C. elegans*, implying that this phenomenon is evolutionarily conserved. In addition, high distributions of three histone modifications (H2BK5me1, H3K36me3 and H4K20me1) as well as RNAPII were observed on pre-miRNA genomic sequences corresponding to potentially transcribed miRNAs and expressed miRNAs.

Nucleosome formation potentially depends significantly on the GC content of DNA sequence [29-31]. Consistently, Ozsolak et al. observed that GC content within pre-miRNA genomic sequence is significantly higher than the surrounding regions [16]. Moreover, we found that the high distributions emerge in all pre-miRNA genomic sequences located in different positions relative to protein-coding genes (intronic, intergenic, and exonic pre-miRNAs). In addition, we found a high correlation between GC content and nucleosome occupancy within pre-miRNA genomic sequence. These facts suggest GC content could be one of the potential factors that influence nucleosome occupancy in pre-miRNA genomic sequence.

Our results revealed chromatin structure characteristics of pre-miRNA genomic sequences. Furthermore, molecular mechanisms should be required to recognize the characteristics, whereas some of the recognition mechanisms just reside in the cleavage mechanisms, since the recognition of pre-miRNA for organism denotes cleavage of pre-miRNA to some extent. There are two possible mechanisms for chromatin structure to improve miRNA cleavage. First, the presence of a positioned nucleosome within the pre-miRNA genomic sequence can act as 'speed bump' to reduce the rate of RNAPII elongation, thus improving pre-miRNA cleavage. Our analyses revealed that nucleosome occupancy is significantly higher in regions of the genome corresponding to the pre-miRNAs, also, we found that there exists enrichment of RNAPII within pre-miRNA, consistently with previous results in several specific miRNAs of Hela cell line [19]. This enrichment could result from the reduction of RNAPII elongation rate caused by nucleosome bound on pre-miRNA. Furthermore, the reduction of RNAPII elongation rate has been shown to promote splicing [36]. Also, due to the rate reduction, the nascent transcript could retain at DNA template longer. Pawlicki et al. indicated that the retention at chromatin could facilitate pri-miRNA processing [19]. Our results showed that the nucleosome occupancies in the genomic sequences of active-promoter miRNAs and expressed miRNAs are significantly higher than the inactive-promoter miRNAs and unexpressed miRNAs. These results suggest that nucleosomes bound on pre-miRNA genomic sequence could slow the elongation rate of RNAPII, thereby improving pre-miRNA cleavage. Similar phenomenon has been reported in some recent studies in splicing site selection for protein-coding genes [25,26,40]. In those studies, nucleosome enrichment is found within exons, compared to introns, and this enrichment within exons can slow the elongation rate of RNAPII to promote splicing. In our studies, we further made a comparison between the nucleosome occupancies within pre-miRNA and exons, and the corresponding occupancy patterns are similar. This comparison strengthens our hypothesis. However, we did not find a significant correlation between nucleosome occupancy and RNAPII level. This is probably because RNAPII level within pre-miRNA genomic sequence is determined not only by nucleosome occupancy, but also by the transcription level of corresponding miRNA. Thus, their relationship can be evaluated, only when the transcription influence is excluded. Similarly, the correlation was not observed between nucleosome occupancy in pre-miRNA genomic sequence and corresponding mature miRNA expression level, consistently with the previous study [16]. This is probably because miRNAs are regulated at multiple levels through their biogenesis [41]. Particularly, pre-miRNA is processed by RNase III endonuclease Dicer to generate the mature miRNA. This process takes place in cytoplasm, which is not influenced by the chromatin structure. More detailed analyses and relevant dataset publication are required to address the relationship between nucleosome occupancy, RNAPII and miRNA expression levels.

Second, some specific histone modifications on pre-miRNA genomic sequence may contribute to pre-miRNA cleavage. Our results showed that there exist enrichments of three specific histone modifications (H2BK5me1, H3K36me3 and H4K20me1) on pre-miRNA genomic sequences corresponding to active-promoter miRNAs and expressed miRNAs. Consistently, several studies have demonstrated that these three histone modifications are the robust transcriptionally active characteristics [22,28,42-44], whereas miRNA cleavage recently is indicated to take place co-transcriptionally [19,20]. Therefore, we hypothesize that these specific features may influence the pri-miRNA cleavage. In addition, numerous Microprocessor-associated proteins were identified as splicing factors [45,46]. Kornblihtt indicates that diverse splicing factors associate with a carboxy terminal domain (CTD) of RNAPII [47], whereas phosphorylated CTD of RNAPII can recruit Histone-lysine N-methyltransferase SETD2, which modulates the histone modification H3K36me3 [48]. Thus, H3K36me3 may interact with Microprocessor via the mediation of CTD, thus influencing the pre-miRNA cleavage. Moreover, Morlando et al. found an enrichment of Drosha in pre-miRNA, and this enrichment correlates with an enrichment of RNAPII [19]. Considering the cross-talk between H3K36me3 and RNAPII mentioned above, this fact also suggests that specific histone modifications may contribute to pre-miRNA cleavage. Recent study indicated that H3K36me3 can interact with Polypyrimidine tract-binding protein (PTBP) [49], and moreover, the change of PTBP levels can differentially influence specific miRNA expression [50]. These facts further validated our hypothesis.

Recently, several studies have been carried out which suggest that exons carry enrichments of nucleosome positioning and specific histone modifications [25,26,40,51], implying that chromatin structure can also modulate exon selection in the process of mRNA maturation. In these studies, similar mechanisms to our study are pointed out. Some similarities can be found between mRNA maturation and miRNA biogenesis. 1) At the DNA level, both of transcriptions rely on RNAPII. 2) At the RNA level, pre-mRNA processing takes place in a large complex known as Spliceosome [52], whereas Spliceosome and Microprocessor share numerous key components [45,53,54]. 3) Recent researches demonstrate that pre-miRNAs are also generated co-transcriptionally, similar to protein-coding genes [19,20]. These similarities also suggest that nucleosome bound on pre-miRNA and exons could share the similar mechanism for splicing.

Some groups indicated that introns carry low nucleosome occupancy, histone modification, and GC content [25,26,40]. However, our results showed that miRNA-coding intron has an enrichment of nucleosome, and this nucleosome carries specific histone modifications, such as H2BK5me1, H3K36me3 and H4K20me1. In addition, we found that GC content is much higher within intronic pre-miRNAs than the flanking regions in the corresponding introns. These differences may be critical to the biogenesis of intronic pre-miRNAs, since both of the two possible mechanisms of pre-miRNA cleavage mentioned above depend on nucleosome occupancy and specific histone modification. Moreover, many studies suggested that mature miRNAs and mRNAs derive from a common nascent transcript [19,39,55]. Consequently, the cleavage of pre-miRNA is possible to influence exon splicing. Given that intronic pre-miRNA is cleaved by Drosha with the help of chromatin structure, the exposed host intron transcript will be rapidly degraded by RNA exonucleases, thus enhancing the exon splicing efficiency [19]. Thus, due to the nucleosome enrichment within intronic pre-miRNA, the corresponding splicing signals may refrain from disruption.

A pioneering study by Ozsolak et al. discovered the nucleosome enrichment on pre-miRNA genomic sequence [16], suggesting that pre-miRNA has specific chromatin structure characteristics. However, the characteristics reside in the DNA level, whereas the cleavage of pre-miRNA occurs in the RNA level. Thus, a confusing problem is raised, how these chromatin structure characteristics can be recognized. Recent study indicated these two levels are coupled for pre-miRNA cleavage [19]. Inspired by the facts above, a series of analyses was performed in this paper. Enrichments of three specific histone modifications as well as RNAPII were also observed on pre-miRNA genomic sequences corresponding to active-promoter miRNAs and expressed miRNAs. These facts together with relevant analyses implied an RNAPII-mediated mechanism that recognizes the chromatin structure characteristics, and links up DNA and RNA levels, thus improving pre-miRNA cleavage.

## Conclusion

MiRNAs are non-coding RNAs with important roles in regulating gene expression. Traditionally, the sequence and chromatin structure are thought to function separately, sequence at RNA level, and chromatin structure at the DNA level. However, accumulating evidence suggests that processes occurring at these two levels are coupled. Recent research suggests that Drosha cleavage of pre-miRNAs from pri-miRNAs occurs co-transcriptionally. However, little is known about the relationship between the chromatin structure and cleavage of pre-miRNA from pri-miRNA. By analysis of genome-wide nucleosome positioning data sets from human and *C. elegans*, we found an enrichment of positioned nucleosome in pre-miRNA, which is highly correlated with GC content within pre-miRNA. In addition, by analysis of genome-wide histone modification data sets from human, we discovered obvious enrichments of three histone modifications (H2BK5me1, H3K36me3 and H4K20me1) on pre-miRNA genomic sequences corresponding active-promoter miRNAs and expressed miRNAs. Moreover, we found enrichment of RNAPII in pre-miRNA genomic sequence. Our results revealed the chromatin structure characteristics of pre-miRNA genomic sequences, and imply potential mechanisms to recognize the characteristics, thus improving pre-miRNA cleavage. There are two possible explanations. First, the presence of a positioned nucleosome within the pre-miRNA can act as 'speed bump' to reduce the rate of RNAPII elongation to improve pre-miRNA cleavage. Second, some specific histone modifications on pre-miRNA may contribute to pre-miRNA cleavage by recruiting Microprocessor. The crucial factors for pre-miRNA recognition are previously understood to only lie in the sequence (RNA level), but our results suggest that they may also reside in the chromatin structure (DNA level), thus providing a new way to better appreciate the connection between chromatin structure, sequence and pre-miRNA cleavage.

## Methods

### Pre-miRNA annotation and miRNA expression profile

We downloaded the annotation of 715 human pre-miRNAs from miRBase [24] and human refGene from the UCSC genome browser [56] (both relative to the hg18 version of the human genome). By comparing the positions of pre-miRNAs obtained from miRBase with the positions of exons obtained from refGene, we obtained intronic, intergenic and exonic pre-miRNAs. In addition, we derived the mature miRNA expression profile and putative miRNA promoters of CD4+ T cells from Ref. [18]. These promoters are predicted employing characteristic transcriptionally active features (RNAPII, H3K4me3 and H2A.Z obtained from [22]), which are the indirect reflection of miRNA transcription. According to mature miRNA expression and miRNA promoter prediction, we obtained four groups of miRNAs, expressed miRNAs, unexpressed miRNAs, active-promoter miRNAs and inactive-promoter miRNAs. miRNAs are expressed, if reads by deep sequencing corresponding to miRNAs are detected; otherwise, miRNAs are unexpressed. Active promoters of miRNAs are identified in 250,000-nt upstream of pre-miRNAs, if all three enrichments of RNAPII, H3K4me3 and H2A.Z are observed, and enrichment of RNAPII co-localizes with the one of H2A.Z and/or H3K4me3, or else, enrichment of H3K4me3 co-localizes with the one of H2A.Z in the upstream regions of the miRNA genomic sequences; otherwise, if miRNAs are unexpressed, and corresponding active promoters are not identified, they are treated as being with inactive promoters. The promoter prediction and enrichment identification of RNAPII, H3K4me3 and H2A.Z refer to [18].

### Nucleosome and histone modification data

We downloaded three datasets of nucleosome positioning and histone modifications from [21-23] (relative to the hg18 version of the human genome). We also obtained genomic sequence of *C. elegans *from the UCSC table browser. Adjusted coverage of nucleosome occupancy in *C. elegans *was obtained from [39] (relative to the ce6 version of the *C. elegans *genome).

We aligned the centers of every pre-miRNA genomic sequences, and defined the center as position zero, downstream positions as plus, and upstream positions as minus. We calculated the scores by assigning each 10-nt sliding window the average of the scores mapping to 140-nt surrounding this window.

ChIP-seq reads for 38 histone modifications in human T cells, and ChIP-seq data for RNAPII were obtained from Refs. [22,23].

**Figure 6:**
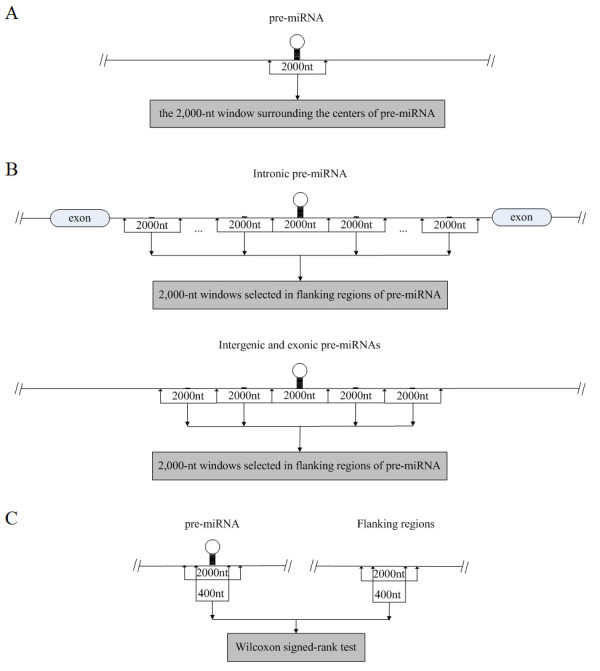
**The window selection for the comparison of histone modification**. (A) The selection of the 2,000-nt window surrounding the center of pre-miRNA genomic sequence. (B) The selection of the 2,000-nt window in the flanking regions of pre-miRNA genomic sequence. (C) The statistical significance of the difference between the histone modification levels within 400-nt windows was evaluated by the Wilcoxon signed-rank test.

To assess whether the enrichment of specific histone modification within pre-miRNA is significant compared to its flanking regions, we made a comparison between two kinds of segments:

1. 2,000-nt windows surrounding the centers of pre-miRNA genomic sequences. We aligned these windows according to the centers of pre-miRNAs, defined the center as position zero, downstream as plus, and upstream as minus. (refer to Figure 6A for details)

2. 2,000-nt windows in the flanking regions of pre-miRNA genomic sequences. We chose different regions for intronic, intergenic and exonic miRNAs, respectively. For intronic miRNA, select every 2,000 nucleotides as a window, from the boundary of the center window above to the splicing sites of the host intron. For intergenic and exonic miRNAs, select two 2,000-nt windows away from the two boundaries of the center window, respectively. (refer to Figure 6B for details)

We made comparison between the nucleosome occupancies within 400-nt windows surrounding the centers of these two segments. The statistical significance of the difference was evaluated by the Wilcoxon signed-rank test (refer to Figure 6C for details). Also, we calculated the mean nucleosome occupancy within a 400-nt window surrounding the center of each miRNA, which is presented in Additional file 2. Based on this dataset, we respectively employed Kendall's rank correlation test to evaluate the correlation between nucleosome occupancies on pre-miRNA genomic sequences and the corresponding mature miRNA expression profiles, and utilized Wilcoxon signed-rank test to make comparison between nucleosome occupancies on pre-miRNA genomic sequences corresponding to miRNAs experiencing different phrases in biogenesis (potentially transcribed, untranscribed, expressed and unexpressed miRNAs) and located in different positions relative to protein-coding genes (intronic, intergenic and exonic miRNAs).

### Comparison between nucleosome occupancy on pre-miRNAs, exons and introns

To make comparison between nucleosome occupancy on pre-miRNA, exon and intron, we summed up the nucleosome occupancy on every pre-miRNA, exon and intron, respectively. Since the lengths of pre-miRNA, exon and intron are different, we divided the sum of nucleosome occupancy of every pre-miRNA, exon or intron by its length, respectively, to obtain the corresponding distributions of nucleosome occupancy for each nucleotide. The statistical significance of the difference between nucleosome occupancies on pre-miRNA, exon and intron was evaluated by the Wilcoxon signed-rank test.

### GC content

GC content of sequence *S *is calculated as:(1)

where *N *is the number of nucleotide *G *or *C *in *S*, and *T *is the total number of nucleotides in *S*. We assigned score of position *i *the GC content of a 100-nt window surrounding this position. The calculation of GC dinucleotides, AT and AT dinucleotides is similar to that of GC content.

To observe the correlation between GC content and nucleosome occupancy, we carried out the following work. 1) Selected the same group of segments utilized for assessing the difference of histone modifications between pre-miRNA and its surrounding regions (which is mentioned in the "Nucleosome and histone modification data" subsection). 2) Calculated the GC-scores within a 400-nt window surrounding the centers of segments. 3) According to the means of the GC-scores, divided each group of segments into five equal bins. 4) Calculated the nucleosome occupancy in each bin.

We used the R language (R Development Core Team 2008) to perform all analyses, using standard methods and packages.

## Abbreviations

miRNA: microRNA; RNAPII: RNA Polymerase II; pri-miRNA: primary miRNA; pre-miRNA: precursor miRNA; nt: nucleotides; H2BK5me1: monomethylation of H2BK5; H3K4me3: trimethylation of H3K4; H3K36me3: trimethylation of H3K36; H4K20me1: monomethylation of H4K20; *C. elegans*: *Caenorhabditis elegans; *TSS: transcription start site; CTD: carboxy terminal domain; PTBP: Polypyrimidine tract-binding protein.

## Authors' contributions

SZ collected the data, designed computational experiments, and carried out statistical analysis. QJ participated in performance of statistical analysis. GW participated in the design of the study. SZ, QJ, BL and YW wrote the manuscript. SZ, GW and MT participated in the revision of this manuscript. YW gave comments and revisions to the final version of this manuscript. All authors read and approved the final manuscript.

## Supplementary Material

Additional file 1 **The percentage of miRNAs with different levels of nucleosome occupancy**. The X axis represents the mean value of nucleosome occupancy in a 400-nt window surrounding the center of pre-miRNA genomic sequence. The y axis represents the percentage of miRNAs with corresponding nucleosome occupancy. Click here for file

Additional file 2 **A detailed annotation for each miRNA together with corresponding expression profile and nucleosome occupancy**. It provides miRNA group annotation (intronic, intergenic, and exonic miRNAs), mature expression profiles, predicted promoters as well as the mean nucleosome occupancy within a 400-nt window surrounding the center of each pre-miRNA genomic sequence. "noData" represents that no data is available for this item. "unidentified" represents that the promoter for this miRNA is not identified. Click here for file
