# Supplementary figures and images for: Chromatin structure characteristics of pre-miRNA genomic sequences
Source: BMC Genomics. 2011 Jun 25;12:329. doi: 10.1186/1471-2164-12-329 (PMC3135579; doi:10.1186/1471-2164-12-329)

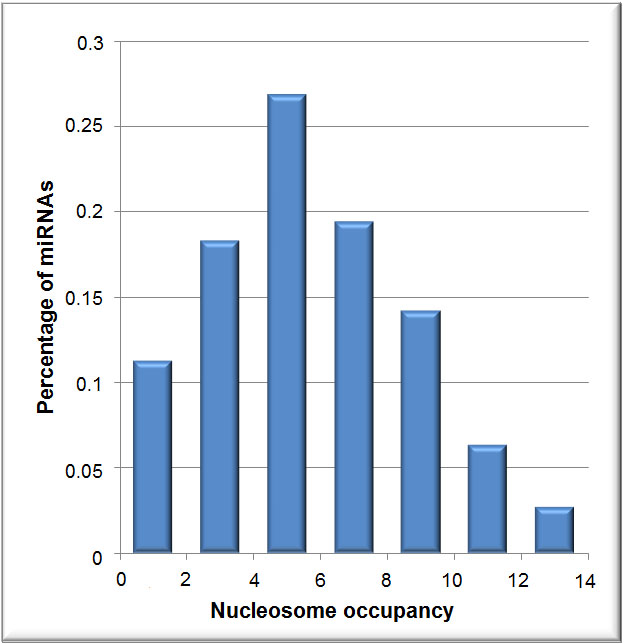

Supplement: Additional file 1 — The percentage of miRNAs with different levels of nucleosome occupancy. The X axis represents the mean value of nucleosome occupancy in a 400-nt window surrounding the center of pre-miRNA genomic sequence. The y axis represents the percentage of miRNAs with corresponding nucleosome occupancy. [file 1471-2164-12-329-S1.JPEG]
